# Assessment of Pupil Size and Angle Kappa in Refractive Surgery: A Population-Based Epidemiological Study in Predominantly American Caucasians

**DOI:** 10.7759/cureus.43998

**Published:** 2023-08-23

**Authors:** Qiancheng Wang, Isabella M Stoakes, Majid Moshirfar, Devon H Harvey, Phillip C Hoopes

**Affiliations:** 1 Medicine, Baylor College of Medicine, Houston, USA; 2 Osteopathic Medicine, Pacific Northwest University of Health Sciences, Yakima, USA; 3 Corneal and Refractive Surgery, Hoopes Vision Research Center, Draper, USA; 4 Ophthalmology and Visual Sciences, John A. Moran Eye Center, University of Utah, Salt Lake City, USA; 5 Eye Banking and Corneal Transplantation, Utah Lions Eye Bank, Murray, USA; 6 Medicine, The Ohio State University College of Medicine, Columbus, USA; 7 Ophthalmology, Hoopes Vision Research Center, Draper, USA

**Keywords:** aberration, photopic, mesopic, smile, prk, lasik, refractive surgery, angle kappa, pupil size, nidek

## Abstract

Purpose

This retrospective study aims to establish normative values for pupil size, angle kappa, higher-order aberration, and astigmatism type in a largely Caucasian population in Utah, United States, utilizing the NIDEK OPD-Scan III system (Gamagori, Japan).

Methods

This study included 716 patients (1432 eyes) grouped based on spherical equivalence and age. Measurements were conducted under mesopic and photopic conditions. Statistical analysis involved Pearson's correlation and linear regression using the generalized estimating equation. NIDEK OPD-Scan III measured mesopic and photopic pupil size and angle kappa. The subjects were then grouped based on their spherical equivalence in diopters (D) and age in decades. The spherical equivalence groups were defined: >-6 D, -5.99 to -3 D, -2.99 to -0.25 D, -0.24 to 0.24 D, and >0.25 D (range 0.25-5.75 D). The higher-order aberration groups were based on the reason for the visit: laser-assisted in situ keratomileusis, photorefractive keratectomy, and small incision lenticule extraction as one group; cataract evaluation; and keratoconus. Astigmatism measurements were grouped into with-the-rule (WRT), against-the-rule (ATR), and oblique astigmatism, with further subgrouping into a young cohort (20-40 years) and an old cohort (>65 years).

Results

Among 716 participants, 49.2% were men; the mean age was 42.1±15.5 (range 7-88 years). The average spherical equivalence for myopia eyes was -3.28±2.34 D, and 1.51±1.46 D for hyperopia eyes. The mean mesopic pupil size was 5.68 ± 1.09 mm; the photopic pupil size was 4.65±1.09 mm. Pearson’s correlation coefficient for mesopic pupil size versus age was -0.551, and -0.42 for photopic pupil (p < 0.001); sphere vs mesopic pupil size was -0.200, and -0.173 for photopic pupil (p < 0.001). The regression analysis for mesopic pupil size versus age revealed a 0.39 mm decrease in average pupil size per decade increase in age, and 0.25 mm decrease per decade for photopic pupil. The regression analysis for mesopic pupil size versus sphere revealed a 0.22 mm decrease in average pupil size per 3D increase in sphere, and a 0.16 mm decrease 3 D increase in sphere for the photopic pupil. The mean mesopic angle kappa was 0.33 ± 0.15 mm; photopic angle kappa was 0.31±0.15 mm. Pearson's correlation coefficient for mesopic angle kappa vs spherical equivalence was 0.32, and 0.296 for photopic angle kappa (p <0.001 for both). Regression analysis for mesopic angle kappa vs spherical equivalence demonstrated a 0.051 mm increase in angle kappa per 3 D increase in spherical equivalence, and a 0.048 mm increase for photopic angle kappa (p < 0.001 for both). Among the higher-order aberration groups, the keratoconus group exhibited the highest levels. In terms of astigmatism type, WRT astigmatism was the most common in the young cohort, while ATR astigmatism was most prevalent in the older cohort.

Conclusions

The results of this study reveal significant associations between pupil size and increasing age, as well as between pupil size and increasingly positive refractive errors. These findings hold particular clinical relevance to older patients and individuals with hyperopia, as they undergo photoablative corneal refractive surgery or multifocal intraocular lens implantation. Understanding the established normative values for pupil size, angle kappa, higher-order aberration, and astigmatism type can aid clinicians in making more informed decisions and improving patient outcomes.

## Introduction

Pupil sizes play a crucial role in assessing and predicting outcomes in photoablative corneal refractive surgeries such as photorefractive keratectomy (PRK), laser-assisted in situ keratomileusis (LASIK), and small incision lenticule extraction (SMILE) as well as lens-based refractive surgeries such as clear lens exchange (CLE) and cataract extraction intraocular lens implantation (CEIOL). Various studies have investigated the impact of pupil sizes on post-operative visual results, with larger pupils being associated with higher amounts of higher-order aberrations (HOAs) [1-3]. For instance, one study revealed a substantial 20- to 40-fold increase in HOAs with pupil dilation from 3 to 7 mm [4]. However, some studies have not found a significant link between larger pupil sizes and greater clinical visual symptoms, especially in long-term post-operative studies [5-8]. Instead, these studies emphasized that ablation depth and ablation zone size were stronger predictors of post-operative visual complaints like halos, glares, and night vision issues. Thus, the role of pupil size in refractive surgery remains a matter of debate, and pupil measurements remain an integral part of all pre-operative workups.

The relationship between larger pupil size and visual complaints has been explored in patients with multifocal IOL implantation and multifocal contact lenses. Larger pupils have been associated with increased glare, halos, and reduced contrast sensitivity [9-12]. However, they also have better near uncorrected visual acuity compared to smaller pupils. Large pupils allow light to enter through multiple foci of the multifocal lens, leading to superimposed images of various focus points [13-14].

Pupil size has been consistently shown to negatively correlate with increasing age in various studies [2, 14-16]. It is also correlated with refractive error, with myopic pupils generally being larger than hyperopic pupils [16,17]. Previous epidemiology studies have also demonstrated variations in mesopic and photopic pupil sizes based on ethnic backgrounds. For example, a German study reported a mean mesopic pupil size of 6.45 ± 0.82 mm, while a large Beijing study found a mean pupil size of 4.08 ± 0.80 mm [16,18]. Although age and testing light conditions may influence pupil size and limit direct comparisons between studies, ethnic backgrounds have also been shown to account for some of the variations in pupil size.

Angle kappa, the angle between the visual axis and the pupil center, is another measurement with significant clinical relevance. Patients with large angle kappa, such as hyperopic patients, may experience alignment errors with pupil-centered photoablation during refractive surgeries [19-21]. Additionally, large angle kappa has been associated with suboptimal outcomes for multifocal IOL implantation due to an increased risk of visual complaints like halos and glare [22]. As with pupil size, previous population studies have demonstrated variations in average angle kappa across different regions of the world [19-21,23].

Given the significant impact of pupil size and angle kappa on the outcomes of corneal and lens-based refractive surgery and the observed variations across continents, our study aims to analyze data from 1432 eyes of refractive surgery candidates who visited our clinic in Utah, United States, specifically focusing on mesopic and photopic pupil size and angle kappa. Notably, we believe this study represents the first epidemiological investigation of its size for a predominantly Caucasian population in the United States. The findings of this research could provide valuable insights into understanding the norms and patterns of pupil size and angle kappa in this population, aiding clinicians in making informed decisions and improving the overall quality of both corneal and lens-based refractive surgical procedures.

## Materials and methods

Methodology

This retrospective study was conducted at Hoopes Vision Research Center in Draper, Utah, United States, spanning the years 2016-2023. Patient records were randomly selected, with the majority being candidates visiting for refractive surgery evaluations. Rigorous scrutiny was applied to all records, and exclusion criteria encompassed prior ophthalmic surgery, complicated surgery, history of uveitis, history of neurological disorders, and poor imaging quality. The study included 716 subjects (49.2% male, 50.8% female), with a mean age of 42.1 ± 15.5 years. This study was approved by The Biomedical Research Alliance of New York (BRANY) Institutional Review Board (#A20-12-547-823). This study adhered to the tenets of the Declaration of Helsinki, and the Hoopes Vision Ethics Committee approved the consent procedure of this study.

Measurements

Refractive measurements, including sphere and cylinder, mesopic and photopic pupil sizes, angle kappa, and higher-order aberrations, were obtained using the NIDEK OPD-SCAN III system (Gamagori, Japan). Pupil images were captured while patients focused on a distant target. Mesopic pupil size was initially measured under low background lighting (1.0 lux), followed by photopic pupil size measurement (40.2 lux) using a bright flash produced by the OPD-SCAN III system. Angle kappa was quantified as the distance in mm between the pupillary center and the center of alignment light/fixation by the OPD-SCAN III system. Additionally, the system recorded the vector of change between the photopic and mesopic pupil centers, documenting the distance in millimeters and angle of travel, serving as a proxy for the delta angle kappa.

Statistical analysis

Data compilation was carried out using Microsoft Excel (Microsoft Corporation, Redmond, Washington, United States), and statistical analysis was performed using IBM SPSS Statistics for Windows, Version 29.0.1.0 (Released 2023; IBM Corp., Armonk, New York, United States).

Pupil size

For mesopic and photopic pupil size analysis, three independent variables, namely age, spherical refraction, and cylinder refraction, were considered. Descriptive statistics were conducted for all variables, and the generalized estimating equation (GEE) was employed to account for potential inter-eye correlations, allowing the inclusion of both eyes in the study. Pearson's R was utilized for correlations between pupil size and each variable. Multivariate analysis of variance (ANOVA) was performed, and independent two-tailed t-tests were conducted to assess the statistical significance of pupil size and each variable. Linear regression using the GEE was performed to investigate the relationship between age and refractive error with pupil size. To further explore differences in pupil size, eyes were grouped based on age brackets and corrected refractive error. Age was categorized into seven brackets by decades, except for the youngest and oldest groups due to limited sample size (7-20, 21-30, 31-40, 41-50, 51-60, 61-70, 71-88). Additionally, age was separated into a group of 20-45 years old and a group older than 65 years. Subsequently, the study aimed to examine differences in pupil size between populations undergoing PRK, LASIK, SMILE, and CEIOL

Spherical equivalence was classified into groups as follows: high myopia (>-6 diopters (D)), moderate myopia (-5.99 to -3D), mild myopia (-2.99 to -0.25 D), mixed astigmatism (-0.24 to 0.24 D), and hyperopia (0.25 to 5.75 D). A pairwise comparison of means utilizing the GEE was conducted between the groups.

Angle kappa

Similarly, the GEE was employed to facilitate the inclusion of both eyes in the study. Pearson's R, independent t-tests, and linear regression using the GEE were performed to explore the relationship between angle kappa and sphere. Eyes were grouped based on corrected refractive error as previously defined, and a pairwise comparison of means using the GEE was performed to compare the angle kappa of each group. Delta angle kappa was calculated as vectors for the right and left eyes to determine the mean and median delta angle kappa for each eye. This calculation was utilized to plot the centroid shift of the angle kappa from mesopic to photopic pupil.

Other findings

Additional calculations were conducted to determine the population statistics of higher-order aberrations in PRK, LASIK, SMILE, CEIOL, and KCN populations. Furthermore, the distribution of WRT, ATR, and oblique astigmatism was determined in the age 20-40 and age > 65 groups.

## Results

The study comprised 716 subjects, totaling 1432 eyes, with a mean age of 42.1 ± 15.5 years, ranging from 7 to 88 years. The study population consisted of 49.2% male and 50.8% female individuals. A significant proportion of the patients visited the clinic for corneal refractive surgery evaluation, with LASIK, PRK, and SMILE being the primary reasons for their visit. The average spherical equivalence for myopic eyes was found to be -3.28 ± 2.34 D, while hyperopic eyes showed an average spherical equivalence of 1.51 ± 1.46D (Table 1).

**Table 1:** General cohort characteristics including age, sex, reason for visit, etc. LASIK: laser-assisted in situ keratomileusis, PRK: photorefractive keratectomy, SMILE: small incision lenticule extraction, D: Diopter

Sex	Male	352 (49.2%)
	Female	364 (50.8%)
Age	Mean	42.1
	Minimum	7
	Maximum	88
Reason for visit	Refractive surgery (PRK, LASIK, SMILE) consult	377 (52.6%)
	Keratoconus evaluation	20 (2.8%)
	Cataract evaluation	70 (9.8%)
	Other (dry eye, glasses, infectious, corneal abrasions, etc)	249 (34.8%)
Refractive error	>-6 D	117 (8.2%)
	-5.99 to -3 D	397 (27.7%)
	-2.99 to -0.25 D	630 (44.0%)
	-0.24 to 0.24 D	70 (4.9%)
	>0.25 D (0.25 to 5.75 D)	218 (15.2%)

Pupil size

The mean mesopic pupil size was found to be 5.68 ± 1.09 mm, whereas the mean photopic pupil size was 4.65 ± 1.09 mm. The average difference in pupil size between mesopic and photopic light conditions was 1.69 ± 0.59 mm. The average sphere and cylinder readings were -2.85 ± 2.96 D and 0.86 ± 0.99 D, respectively. Pearson's R analysis revealed statistically significant negative correlations between mesopic pupil size and age (-0.551) and between photopic pupil size and age (-0.42). Similarly, significant negative correlations were observed between pupil size and sphere (-0.200 and -0.173 for mesopic and photopic pupil size, respectively), and pupil size and cylinder (-0.021 for mesopic pupil size, and -0.056 for photopic pupil size), with all p-values being less than 0.001, except for mesopic pupil size and cylinder (p = 0.223).

**Figure 1:**
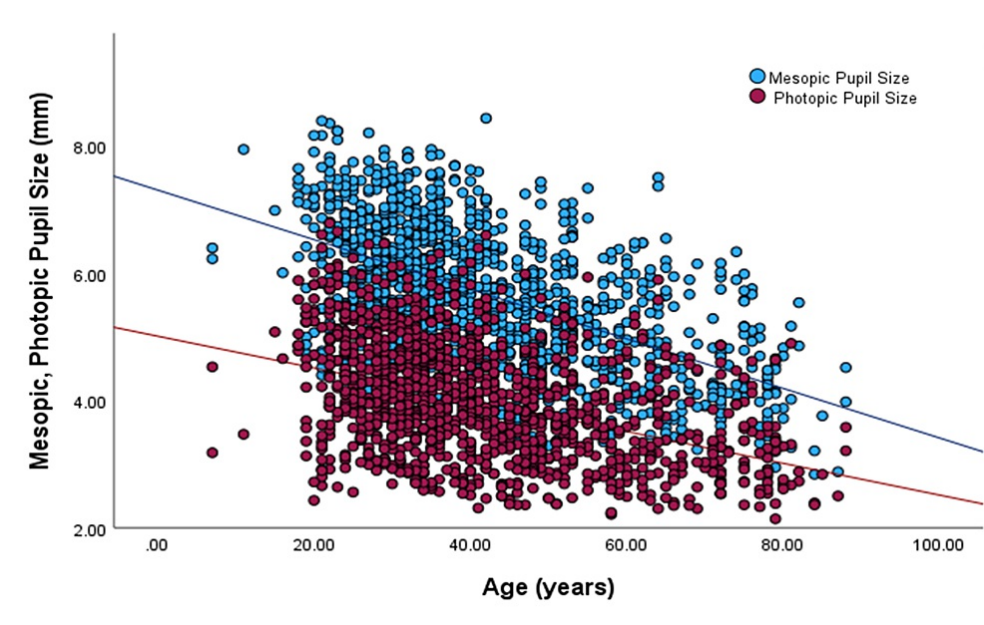
Photopic and mesopic pupil sizes as a function of age Lines represent the linear regression fit (y = -0.025x + 5.013, r = - 0.420, maroon circles; and (y = -0.039x + 7.311, r = - 0.551, blue circles) for photopic and mesopic pupils, respectively.

Linear regression was performed to explore the relationships further. For mesopic and photopic pupil size with age, the best-fit line equations indicated a 0.39 and 0.25 mm decrease in average pupil size per decade increase in age, respectively (Figure 1).

Regarding pupil size and spherical equivalence, the linear regression yielded a 0.22 and 0.16 mm decrease in pupil size per 3 D increase in sphere for mesopic and photopic pupil size, respectively. Additionally, the linear regression analysis for photopic pupil size and cylinder indicated a 0.15 mm decrease in size per 3 D increase in cylinder.

**Figure 2:**
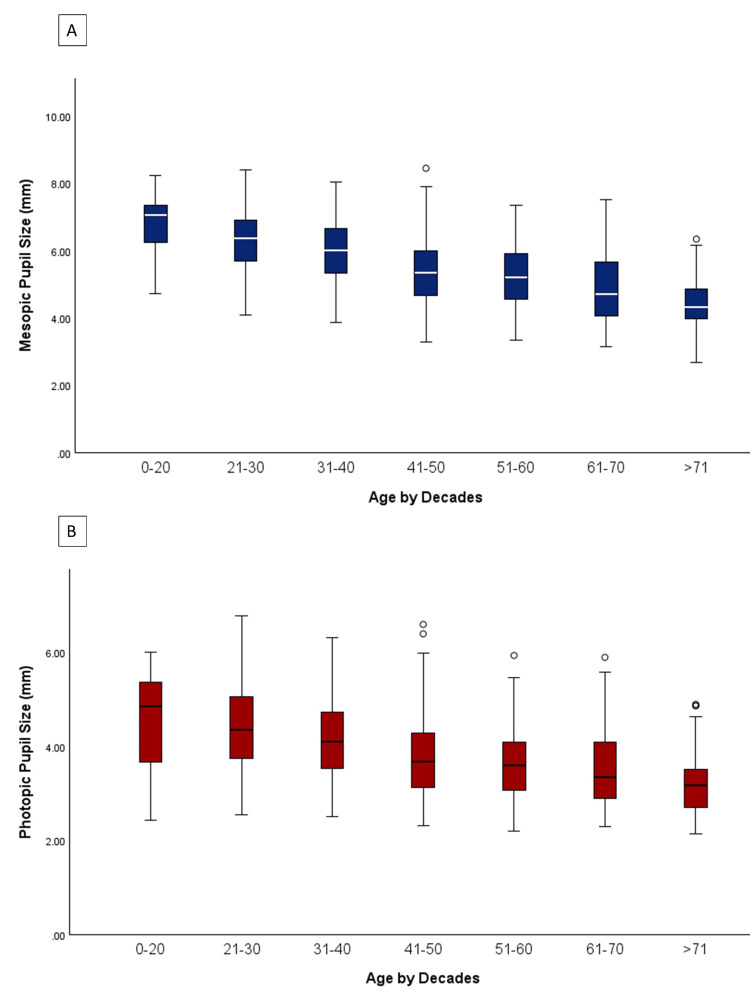
Box and whisker plot of mesopic (A) and photopic (B) pupil size by age brackets

Eyes were systematically grouped into age brackets, and a pairwise comparison of mean pupil size was conducted for both mesopic and photopic pupils. The analysis revealed statistically significant differences among nearly all age brackets (Figure 2).

**Figure 3:**
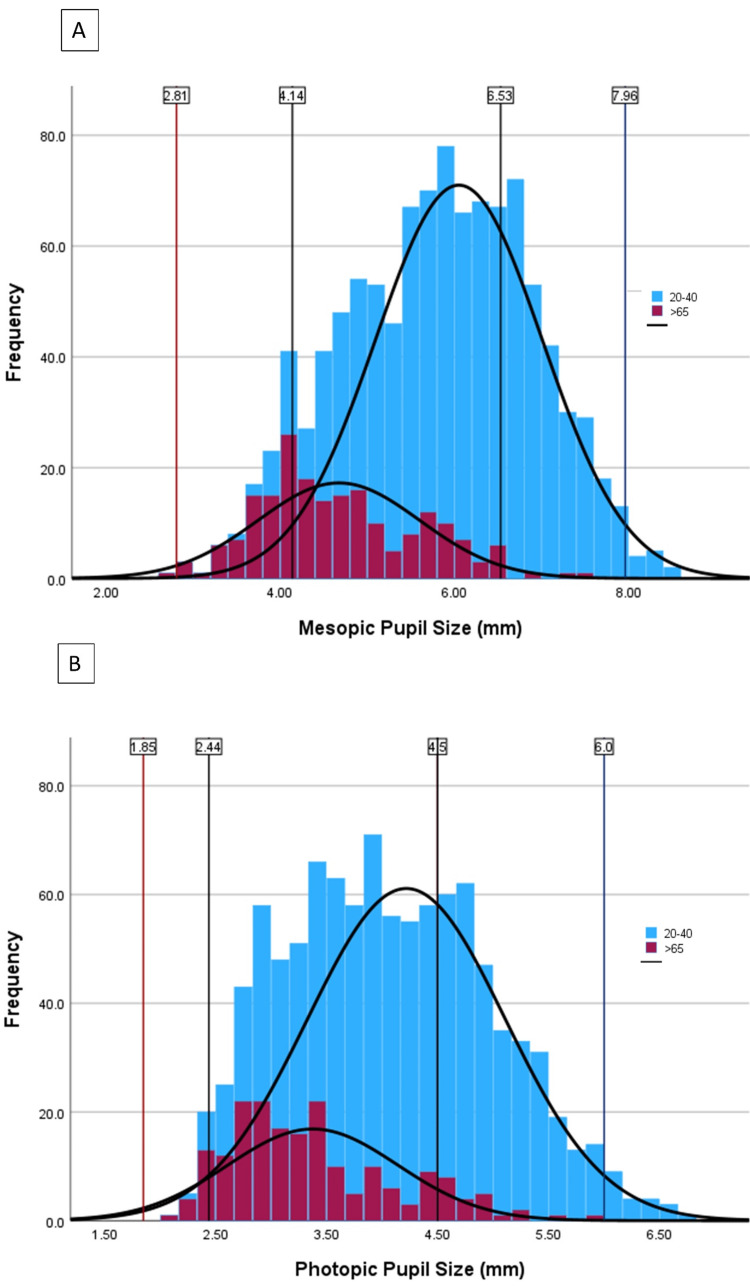
Normative distribution of mesopic (A) and photopic (B) pupil sizes in two age groups Pupil sizes at two standard deviations from the mean are shown by line and data label. Mesopic mean age 20-40: 6.05 ± 0.96; age > 65 4.67 ± 0.93. Photopic mean age 20-40: 4.22 ± 0.89; age > 65: 3.38 ± 0.77.

Additionally, pupil sizes were compared between two specific age groups, namely the 20-40 age bracket and the age >65 age bracket. These two groups represent individuals most likely to seek PRK, LASIK, and SMILE versus CEIOL. The average mesopic pupil size for the 20-40 age group was found to be 6.05 ± 0.96 mm, while the age >65 group had an average mesopic pupil size of 4.67 ± 0.93 mm, showing a statistically significant difference (p < 0.001). Similarly, for photopic pupil size, the 20-40 age group exhibited an average size of 4.22 ± 0.89 mm, whereas the age >65 group had an average size of 3.38 ± 0.77 mm (Figure 3).

In addition to age-based grouping, eyes were categorized based on refractive errors into myopia (<-0.25 D), mixed astigmatism (-0.24 to 0.24 D), and hyperopia (0.25 to 5.75 D) (Table 2).

**Table 2:** Pupil sizes by spherical equivalence group SD: standard deviation.

	Condition	Mean (mm)	SD	Maximum (mm)	Minimum (mm)	Range (mm)
Myopia	Photopic	4.09	0.91	6.79	2.24	4.55
	Mesopic	5.84	1.06	8.44	2.88	5.56
Mixed astigmatism	Photopic	3.51	0.83	5.60	2.14	3.46
	Mesopic	4.94	1.15	7.32	2.67	4.65
Hyperopia	Photopic	3.54	0.76	6.47	2.21	4.26
	Mesopic	5.10	0.93	7.94	3.21	4.73

**Figure 4:**
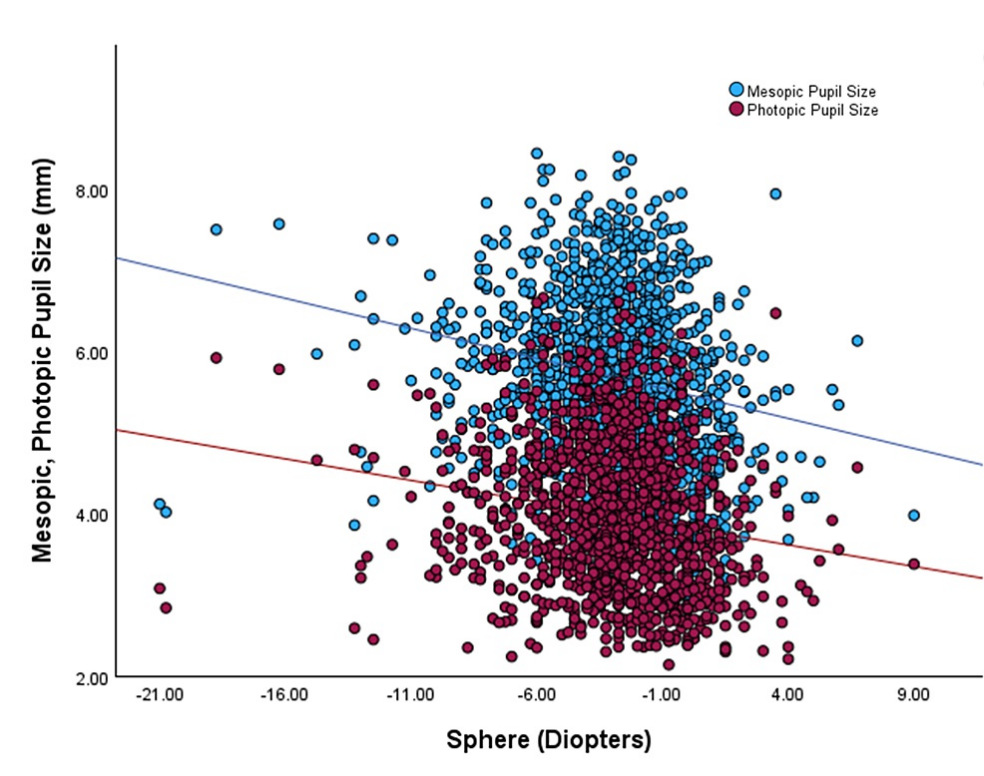
Photopic and mesopic pupil sizes as a function of sphere Lines represent the linear regression fit (y = -0.053x + 3.828, r = -0.173, p < 0.001 maroon circles; and (y = -0.074x + 5.470, r = - 0.2, p < 0.001 blue circles) for photopic and mesopic pupils, respectively.

A pairwise comparison of means showed significant differences between mesopic and photopic pupil sizes in myopic eyes compared to mixed astigmatism and hyperopia eyes (p < 0.001 for both comparisons) (Figure 4).

However, no statistically significant difference was observed between mesopic and photopic pupil sizes in mixed astigmatism and hyperopia eyes (p = 0.789).

For a more detailed analysis of myopic eyes, they were further divided into low myopia (-2.99 to -0.25 D), moderate myopia (-5.99 to -3 D), and high myopia (more than -6 D) groups, and comparisons were made among these groups, as well as with mixed astigmatism and hyperopia eyes. Statistically significant differences were retained for all myopia groups when compared to mixed astigmatism and hyperopia eyes (p < 0.001 for all comparisons). However, there was no statistically significant difference between the myopia groups themselves (high vs. moderate p = 0.782; high vs. low p = 0.445, moderate vs. low p = 0.104).

Angle kappa

Angle kappa is measured as the distance in mm between the pupillary center and the center of fixation. The mean angle kappa for mesopic and photopic pupils was found to be 0.33 ± 0.15 mm and 0.31 ± 0.15 mm, respectively (Table 3).

**Table 3:** Angle kappa by spherical equivalence group SD: standard deviation.

	Condition	Mean (mm)	SD	Maximum (mm)	Minimum (mm)	Range (mm)
High myopia	Photopic	0.2514	0.11762	0.63	0.04	0.59
	Mesopic	0.2595	0.12324	0.52	0.01	0.51
Moderate myopia	Photopic	0.2685	0.1337	0.7	0.04	0.66
	Mesopic	0.2794	0.1435	0.74	0	0.74
Low myopia	Photopic	0.3113	0.1428	0.75	0.01	0.74
	Mesopic	0.3319	0.1416	0.76	0.03	0.73
Mixed astigmatism	Photopic	0.3438	0.1456	0.72	0.1	0.62
	Mesopic	0.3782	0.1564	0.74	0.06	0.68
Hyperopia	Photopic	0.3791	0.1559	0.81	0.03	0.78
	Mesopic	0.4134	0.1528	0.89	0.08	0.81

**Figure 5:**
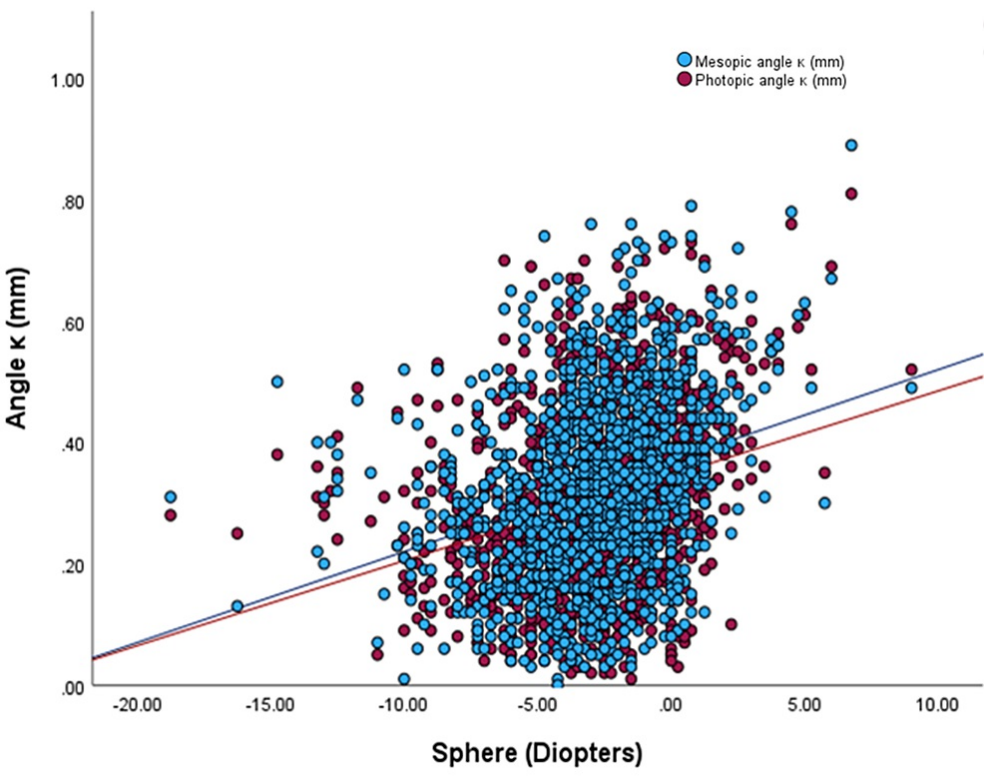
Photopic and mesopic angle kappa as a function of sphere Lines represent the linear regression fit (y= 0.014x + 0.345, r = - 0.272, maroon circles; and (y= 0.015x + 0.370, r = 0.297, blue circles) for photopic and mesopic pupils, respectively.

Pearson's correlation analysis revealed a positive correlation between mesopic and photopic angle kappa with spherical equivalence, with R values of 0.32 and 0.296, respectively (p < 0.001 for both). Linear regression using the GEE was performed, indicating a 0.051 and 0.048 mm change in mesopic and photopic angle kappa per 3 D increase in spherical equivalence, respectively, with both relationships being statistically significant (p < 0.001) (Figure 5).

**Figure 6:**
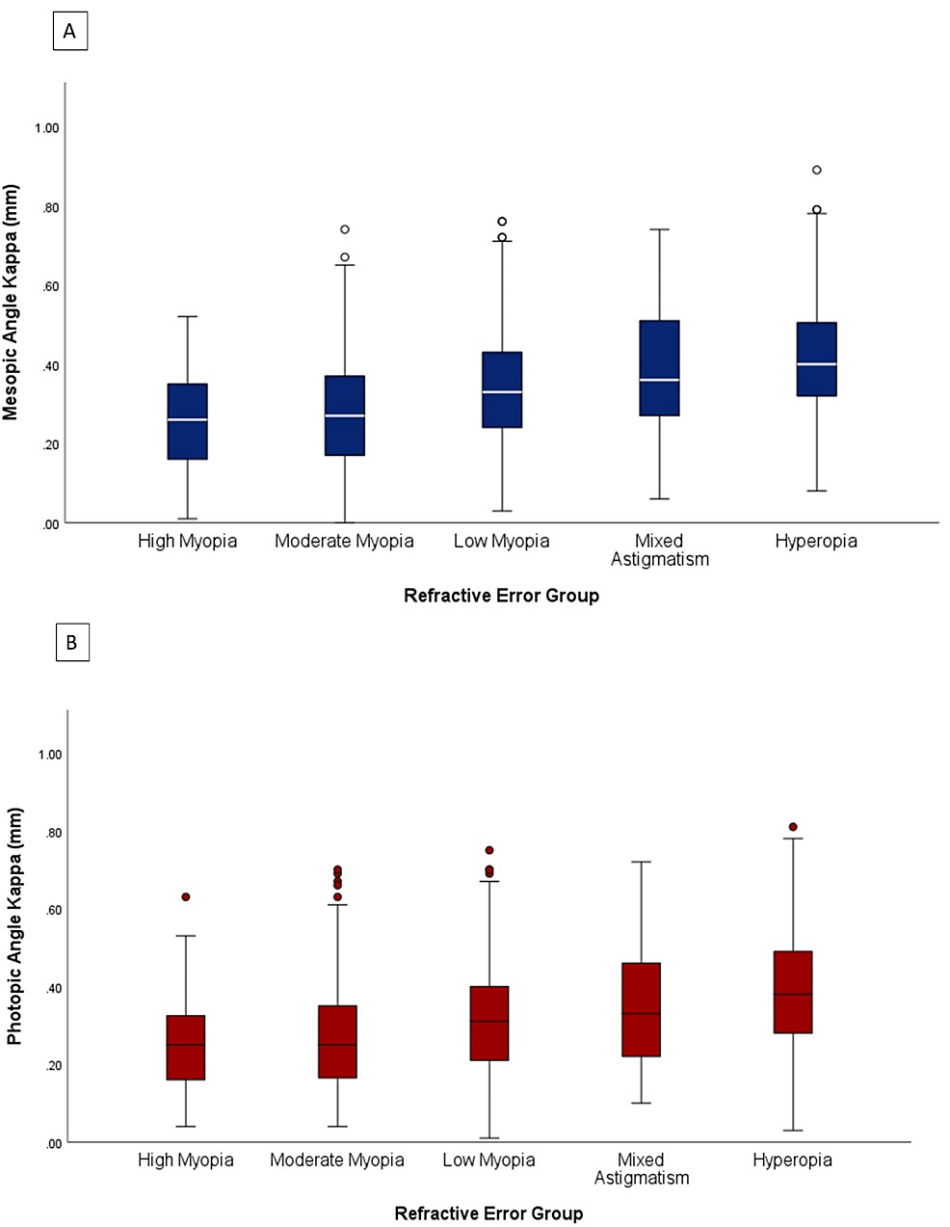
Mesopic (A) and photopic (B) mean angle kappa grouped by refractive error High myopia = sphere > -6 D; moderate myopia = sphere -5.99 to -3 D; low myopia = sphere -2.99 to -0.26 D; mixed astigmatism = corrected refractive error -0.25 to 0.25 D with astigmatism; hyperopia = sphere > 0.26 D.

Angle kappa was further categorized into high myopia (>-6 diopters (D)), moderate myopia (-5.99 to -3 D), mild myopia (<-2.99 to -0.25 D), mixed astigmatism (-0.24 to 0.24 D), and hyperopia (0.25 to 5.75 D) groups. A pairwise comparison of mean photopic and mesopic angle kappa revealed a significant difference between the myopia group versus mixed astigmatism and hyperopia groups (p < 0.001). However, there was no statistically significant difference between mixed astigmatism and hyperopia groups in both mesopic and photopic angle kappa (mesopic p = 0.126, photopic p = 0.132). Low myopia angle kappa was significantly different from high myopia and moderate myopia groups (p < 0.001). On the other hand, there was no statistically significant difference between the high and moderate myopia groups in both mesopic and photopic angle kappa (p = 0.198 and 0.244) (Figure 6).

**Figure 7:**
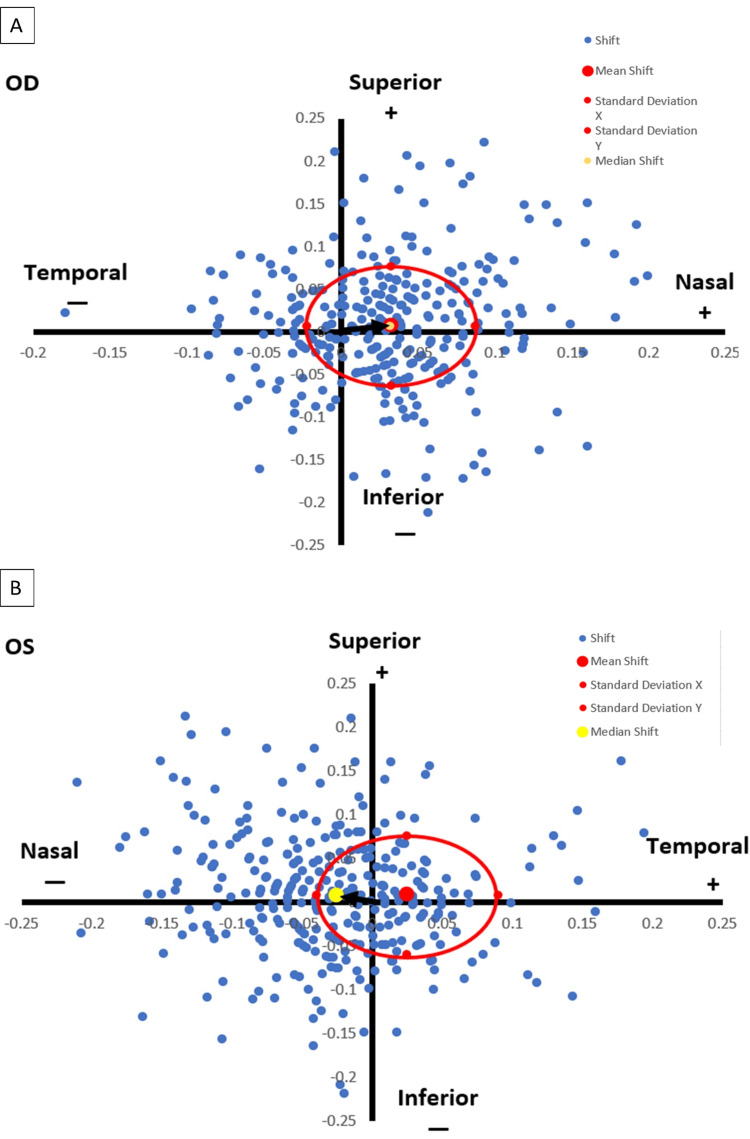
Pupil centroid shift in the right (A) and left eye (B) The circle shows one standard deviation from the mean. The black arrow points from the chart origin to the median of the centroid shift.

The delta angle kappa is assessed as the distance in millimeters between the mesopic and photopic angle kappa at a particular angle. This piece of data is then utilized to compute the vector for delta angle kappa, which is further categorized for the right and left eye. Subsequently, the median and mean angle kappa for each eye was calculated. The centroid shift for delta angle kappa was determined using the above information, and the results are plotted in Figure 7.

Higher-order aberration

Analysis of HOAs was carried out for three distinct patient populations: those seeking corneal refractive surgery (PRK, LASIK, SMILE), CEIOL, and those seeking management for KCN (Table 4).

**Table 4:** HOAs by reason for visit LASIK: laser-assisted in situ keratomileusis, PRK: photorefractive keratectomy, SMILE: small incision lenticule extraction, CEIOL: cataract extraction intraocular lens implantation, KCN: keratoconus, HOA: higher-order aberrations, SD: standard deviation.

	HOA	Mean (μm)	SD	Maximum (μm)	Minimum (μm)	Range (μm)
Refractive surgery (PRK, LASIK, SMILE)	Total	0.32	0.23	3.93	0.05	3.88
	Corneal	0.36	0.28	3.76	0.04	3.72
	Internal	0.34	0.20	1.94	0.06	1.87
CEIOL	Total	0.5	0.34	1.78	0.08	1.70
	Corneal	0.41	0.29	1.52	0.05	1.48
	Internal	0.45	0.28	1.55	0.13	1.43
KCN	Total	1.05	0.84	4.10	0.15	3.95
	Corneal	1.60	1.55	7.38	0.25	7.13
	Internal	0.96	0.93	4.31	0.16	4.15

**Figure 8:**
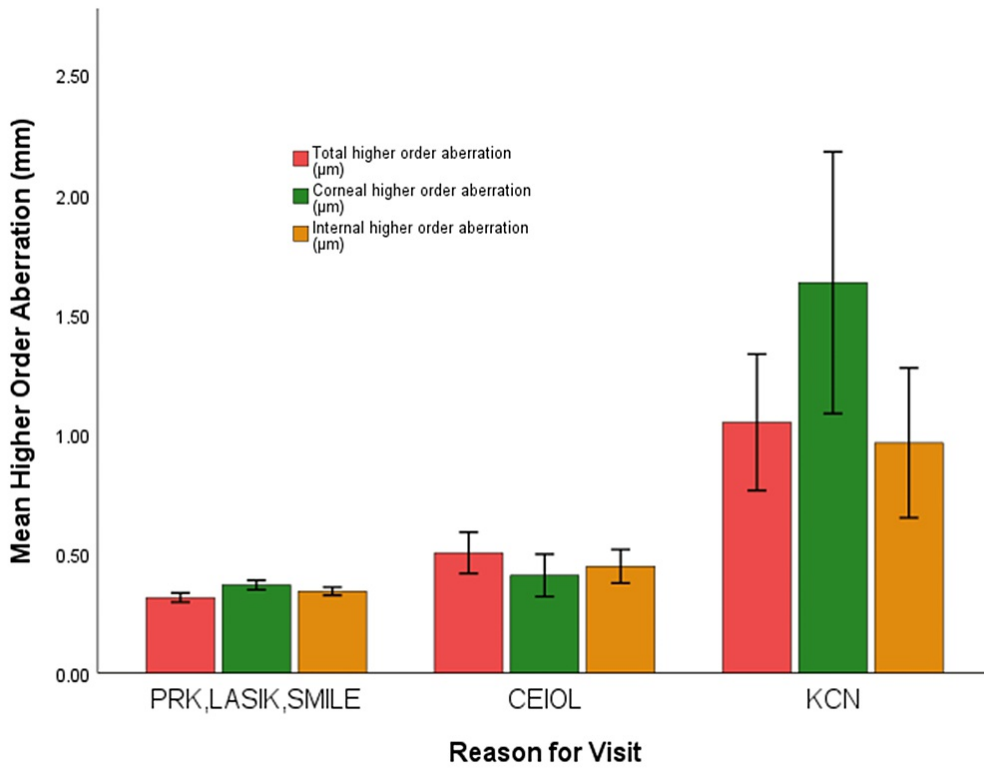
Mean higher order aberration (corneal + internal; corneal; internal) grouped by reason for visit (PRK, LASIK, SMILE; CEIOL; KCN) LASIK: laser-assisted in situ keratomileusis, PRK: photorefractive keratectomy, SMILE: small incision lenticule extraction, CEIOL: cataract extraction intraocular lens implantation, KCN: keratoconus.

Pairwise comparison of the patient groups revealed statistically significant differences in all types of HOAs for the KCN group compared to all other groups (p < 0.001 for all comparisons, except internal HOA KCN vs CEIOL, where p = 0.004). Additionally, internal and total HOAs were found to be significantly different between the CEIOL group and the PRK, LASIK, and SMILE group (p = 0.005 and p < 0.0001, respectively) (Figure 8).

These findings indicate distinct patterns of higher-order aberrations among the three patient groups, highlighting the significance of HOA assessment in different clinical scenarios.

Astigmatism types

**Figure 9:**
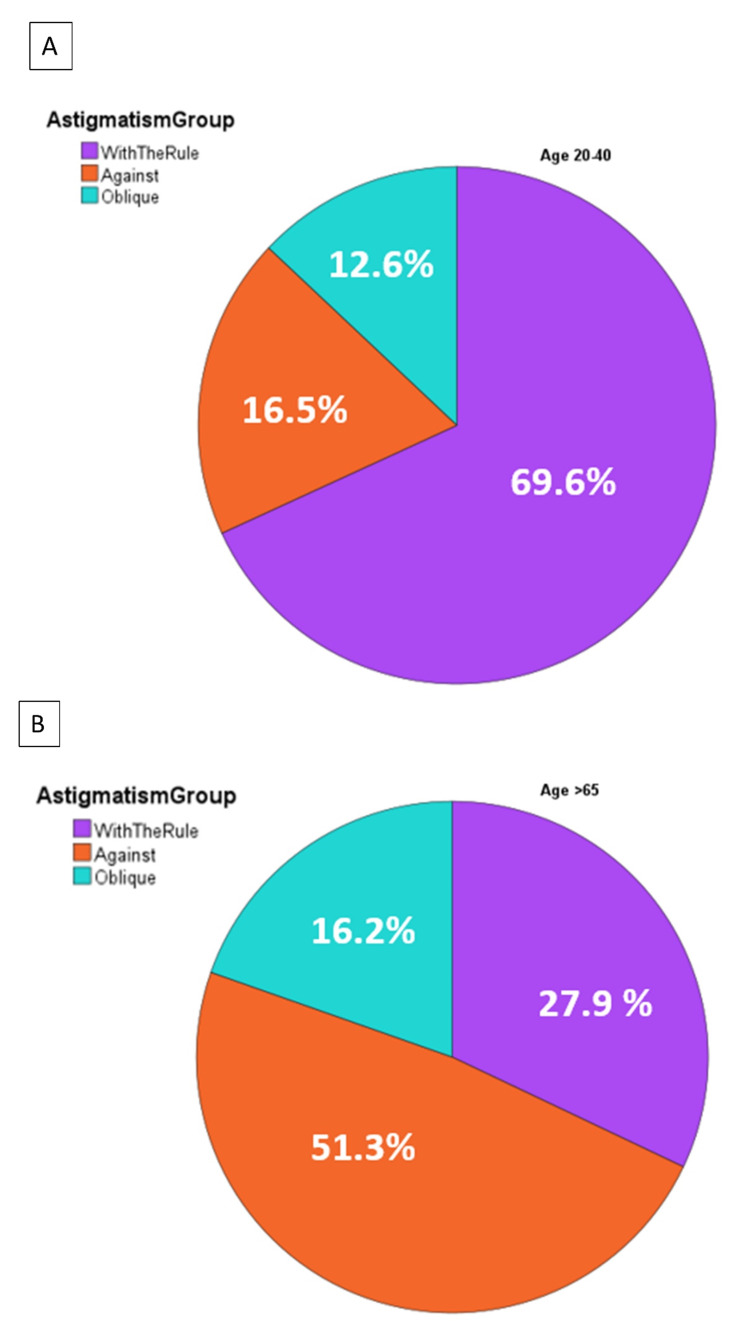
Prevalence of with the rule, against the rule, and oblique astigmatism grouped by age between 20 and 40 and age greater than 65.

The prevalence of different astigmatism types was investigated in two distinct age groups: the young age group (age 20-40) and the old age group (age > 65). The analysis revealed a significantly higher percentage of WTR astigmatism and a lower percentage of ATR astigmatism in the younger age group (p < 0.001) (Figure 9).

These findings highlight the age-related differences in astigmatism patterns, with WTR astigmatism being more prevalent in the younger age group compared to the older age group. 

## Discussion

In this study, we investigated pupil size and angle kappa in a predominantly Caucasian population in Utah, USA. Our findings revealed that the mean photopic and mesopic pupil sizes were 4.65 ± 1.09 mm and 5.68 ± 1.09 mm, respectively. These results are consistent with many previous population studies, but differ from others, which may be attributed to variations in population demographics [16, 18, 24]. Among the studies with similar pupil sizes were populations of Iranian, Chinese, and European descent [14, 25]. For the studies with smaller pupil sizes, one study exclusively focused on a cataract population, while the other was a smaller normative study [14, 26]. We observed a negative correlation between age and pupil size, as well as smaller pupils in hyperopic eyes compared to myopic eyes, aligning with previous epidemiologic research [14, 16, 25]. Furthermore, we found a smaller photopic pupil size associated with greater astigmatism, although this finding was not reflected in mesopic pupil size and astigmatism. The relationship between cylinder and pupil size remains a contentious subject in current studies, with some providing evidence to support a correlation, while others show no such association [16, 18, 23].

When divided into age brackets, the difference in pupil size remained especially apparent. This is particularly evident when comparing eyes in the 20-40 age bracket with those in the >65 age bracket. This holds significant implications for decision-making in corneal refractive surgery, multifocal contact lenses, and multifocal intraocular lens implantation. In the rare event a younger patient may need to receive a multifocal intraocular lens implant, or perhaps that of a CLE, the lens choice would differ significantly from an elderly patient. The difference between the pre-presbyopia (age 30-40) and presbyopia group (40-60) is less apparent. However, the mean mesopic pupil size for the pre-presbyopia group is 0.6-0.8 mm larger than the presbyopia group. Although visual complaints generally decrease with smaller pupil size, special consideration is needed for how the implant lenses will function as the patient ages [5-9].

While larger pupil size has been found to result in greater amounts of HOAs, pupil size has not been shown to cause postoperative visual symptoms such as night vision problems, halos, or glare [1-3, 5, 6]. Instead, these complaints have been associated with small ablation zones without transition zones, greater ablation depths, and residual postoperative spherical equivalence. As anticipated, corneal and total HOAs were higher for the KCN group than for the others. The cataract group exhibited higher internal HOAs compared to the photoablation group. These findings are consistent with other studies, including a population study involving 6311 subjects. Subclinical keratoconus might manifest with more HOAs. In a study encompassing 1488 eyes, the odds ratio for postoperative visual complaints for myopia greater than 5D, optical zone of 6mm or less, and postoperative spherical equivalence of 0.5D or more away from emmetropia was 2.8, 2.5, and 2.9, respectively [5]. Nevertheless, the significance of pupil size in photoablative corneal and lens-based refractive surgery remains controversial, and studies recommend incorporating pupil size into both preoperative and postoperative planning [2, 6].

The mean angle kappa for mesopic and photopic pupils was 0.33 mm ± 0.15 mm and 0.31 mm ± 0.15 mm, respectively. Domínguez-Vicent et al. reported angle kappa values of 0.43 ± 0.13 mm and 0.27 ± 0.15 mm, measured using the Orbscan II and Galilei G4 systems [19]. However, the authors did not specify the lighting conditions under which the angle kappa was measured. Our results align with other studies that indicate a smaller angle kappa in myopic eyes compared to hyperopic eyes [21]. On the other hand, Domínguez-Vicent et al. conducted an analysis on angle kappa within +1D, -1D, -2D, -3D, and -4D groups, yet found no significant difference between these groups. A Korean study involving 436 eyes similarly did not discover a significant difference among the myopia, emmetropia, and hyperopia groups, but did observe the smallest angle kappa in high myopia eyes [23]. However, two other studies did identify a significant difference, with myopic eyes exhibiting a smaller angle kappa [27, 28]. Our study, encompassing over 1400 eyes, potentially holds greater statistical power to detect differences in angle kappa between refractive error groups when compared to smaller studies.

Large angle kappa is also a consideration in multifocal IOLs. The fovea of individuals with larger angle kappa may receive fovea-centric rays from the edge of implant lens rings instead of the center, creating a more photopic phenomenon [29-33]. A study by Prakash et al. found patients with greater angle kappa reported a higher incidence of halos and glare. Similar findings are reported in a study of Chinese patients [34]. In a randomized controlled trial, patients were fitted with angle-kappa customized IOLs. The authors of this study found customized IOLs to improve intermediate visual acuity [35]. Our findings show larger angle kappa in the hyperopia group.

Our study also reports higher-order aberration measured by the OPD Scan III system in eyes that were under evaluation for photoablative corneal refractive surgery (PRK, LASIK, SMILE), cataract surgery, and KCN management. The OPD Scan system has been reported in the measurement of HOAs with reliable results [36,37]. As expected, the corneal and total HOA was higher for the KCN group than others [38]. The cataract group had higher internal HOA than the photoablation group [39]. This corresponds with other studies, including a population study of 6311 subjects [40]. Subclinical keratoconus may present with more HOAs [41]. In our study, the mean corneal HOA was almost four times greater than the cataract and photoablation group (1.59 ± 1.55 μm versus 0.42 ± 0.29 μm and 0.36 ± 0.28 μm, respectively).

The significantly higher percentage of WTR astigmatism in the younger group and the significantly higher percentage of AGR astigmatism in the older group is similar to findings in other population studies [41,42]. Although the cause of this change is unclear, astigmatism appears stable until the fifth decade of life, after which eyes slowly develop ATR astigmatism. Based on the findings of Naeser et al., there is approximately a 0.25 D drift from WTR astigmatism towards ATR astigmatism per decade after the age of 49 [43]. Photoablation refractive surgery in middle-aged patients should take into account the astigmatic changes that are likely to occur.

Strengths, limitations, and future research

As with any epidemiologic study, there are certain limitations to consider. The retrospective design and the potential bias towards a specialized private practice focusing on refractive and cataract surgery could impact the generalizability of our results. Moreover, variations in instrumentation, measurement tools, and lighting conditions across different studies may contribute to the observed differences in measurements. Future epidemiologic investigations encompassing diverse populations across the United States may further elucidate whether these variances are attributed to the unique characteristics of the Utah and US population or specific factors related to our study cohort. Additionally, more specific exclusion criteria could have alleviated additional confounding variables, such as glaucoma, or macular degeneration, especially when including more advanced age groups. Considering the study's strengths and limitations, we propose future research endeavors, including prospective studies with larger and more diverse cohorts, longitudinal assessments to track changes over time, comparison of pupillometry and HOA measurements obtained with iTrace or other machines, and investigations into other potential confounding factors. By pursuing these avenues, we aim to enhance the validity of our findings and contribute further to the understanding of ocular measurements and their implications on clinical outcomes.

## Conclusions

Our study provides valuable insights into pupil size, angle kappa, HOAs, and astigmatism type using the advanced OPD Scan III system. To our knowledge, this represents the largest study on pupil size conducted within a US population to date. The findings of our research align with existing global trends; however, we acknowledge slight variations in specific measurements. The comprehensive data obtained from this study offers valuable information for clinicians, aiding in the decision-making process for corneal and lens-based refractive surgery planning. Additionally, understanding the impact of pupil size, angle kappa, and HOAs on visual outcomes can potentially lead to improved patient satisfaction and better treatment strategies.

## References

[1] Oliver KM, Hemenger RP, Corbett MC, O'Brart DP, Verma S, Marshall J, Tomlinson A (1997). Corneal optical aberrations induced by photorefractive keratectomy. J Refract Surg.

[2] Zhou J, Xu Y, Li M, Knorz MC, Zhou X (2018). Preoperative refraction, age and optical zone as predictors of optical and visual quality after advanced surface ablation in patients with high myopia: a cross-sectional study. BMJ Open.

[3] Queirós A, Villa-Collar C, González-Méijome JM, Jorge J, Gutiérrez AR (2010). Effect of pupil size on corneal aberrations before and after standard laser in situ keratomileusis, custom laser in situ keratomileusis, and corneal refractive therapy. Am J Ophthalmol.

[4] Oshika T, Klyce SD, Applegate RA (1999). Comparison of corneal wavefront aberrations after photorefractive keratectomy and laser in situ keratomileusis. Am J Ophthalmol.

[5] Pop M, Payette Y (2004). Risk factors for night vision complaints after LASIK for myopia. Ophthalmology.

[6] Schallhorn S, Brown M, Venter J, Hettinger K, Hannan S (2014). The role of the mesopic pupil on patient-reported outcomes in young patients with myopia 1 month after wavefront-guided LASIK. J Refract Surg.

[7] Schallhorn SC, Kaupp SE, Tanzer DJ, Tidwell J, Laurent J, Bourque LB (2003). Pupil size and quality of vision after LASIK. Ophthalmology.

[8] Schmidt GW, Yoon M, McGwin G, Lee PP, McLeod SD (2007). Evaluation of the relationship between ablation diameter, pupil size, and visual function with vision-specific quality-of-life measures after laser in situ keratomileusis. Arch Ophthalmol.

[9] Teshigawara T, Meguro A, Mizuki N (2021). The effect of age, postoperative refraction, and pre- and postoperative pupil size on halo size and intensity in eyes implanted with a trifocal or extended depth-of-focus lens. Clin Ophthalmol.

[10] Vega F, Alba-Bueno F, Millán MS, Varón C, Gil MA, Buil JA (2015). Halo and through-focus performance of four diffractive multifocal intraocular lenses. Invest Ophthalmol Vis Sci.

[11] Pieh S, Lackner B, Hanselmayer G (2001). Halo size under distance and near conditions in refractive multifocal intraocular lenses. Br J Ophthalmol.

[12] Knorz MC, Bedoya JH, Hsia TC (1992). Comparison of modulation transfer function and through focus response with monofocal and bifocal IOLs. Ger J Ophthalmol.

[13] Madrid-Costa D, Ruiz-Alcocer J, García-Lázaro S, Ferrer-Blasco T, Montés-Micó R (2015). Optical power distribution of refractive and aspheric multifocal contact lenses: effect of pupil size. Cont Lens Anterior Eye.

[14] Guillon M, Dumbleton K, Theodoratos P, Gobbe M, Wooley CB, Moody K (2016). The effects of age, refractive status, and luminance on pupil size. Optom Vis Sci.

[15] Alfonso JF, Ferrer-Blasco T, González-Méijome JM, García-Manjarres M, Peixoto-de-Matos SC, Montés-Micó R (2010). Pupil size, white-to-white corneal diameter, and anterior chamber depth in patients with myopia. J Refract Surg.

[16] Linke SJ, Baviera J, Munzer G, Fricke OH, Richard G, Katz T (2012). Mesopic pupil size in a refractive surgery population (13,959 eyes). Optom Vis Sci.

[17] Cakmak HB, Cagil N, Simavli H, Duzen B, Simsek S (2010). Refractive error may influence mesopic pupil size. Curr Eye Res.

[18] Wang YX, Xu L, You QS, Yang H, Jonas JB (2013). Pupil size: the Beijing eye study. Acta Ophthalmol.

[19] Domínguez-Vicent A, Monsálvez-Romín D, Pérez-Vives C, Ferrer-Blasco T, Montés-Micó R (2014). Measurement of angle kappa with Orbscan II and Galilei G4: effect of accommodation. Graefes Arch Clin Exp Ophthalmol.

[20] Gharaee H, Shafiee M, Hoseini R, Abrishami M, Abrishami Y, Abrishami M (2015). Angle kappa measurements: Normal values in healthy Iranian population obtained with the Orbscan II. Iran Red Crescent Med J.

[21] Gharieb Ibrahim HM, Gharieb HM, Othman IS (2022). Angle κ measurement and its correlation with other ocular parameters in normal population by a new imaging modality. Optom Vis Sci.

[22] Prakash G, Prakash DR, Agarwal A, Kumar DA, Agarwal A, Jacob S (2011). Predictive factor and kappa angle analysis for visual satisfactions in patients with multifocal IOL implantation. Eye (Lond).

[23] Choi SR, Kim US (2013). The correlation between angle kappa and ocular biometry in Koreans. Korean J Ophthalmol.

[24] Cakmak HB, Cagil N, Simavli H, Raza S (2012). Corneal white-to-white distance and mesopic pupil diameter. Int J Ophthalmol.

[25] Elkitkat RS, Fouad YA, Shams A, Hamza I (2020). Normative values of corneal spherical aberration, pupil size, and other key refractive and topographic parameters in a large cohort of Egyptian cataract surgery candidates. Clin Ophthalmol.

[26] Haw WW, Manche EE (2001). Effect of preoperative pupil measurements on glare, halos, and visual function after photoastigmatic refractive keratectomy. J Cataract Refract Surg.

[27] Basmak H, Sahin A, Yildirim N, Papakostas TD, Kanellopoulos AJ (2007). Measurement of angle kappa with synoptophore and Orbscan II in a normal population. J Refract Surg.

[28] Hashemi H, KhabazKhoob M, Yazdani K, Mehravaran S, Jafarzadehpur E, Fotouhi A (2010). Distribution of angle kappa measurements with Orbscan II in a population-based survey. J Refract Surg.

[29] Frings A, Druchkiv V, Pose L, Linke SJ, Steinberg J, Katz T (2019). Analysis of excimer laser treatment outcomes and corresponding angle κ in hyperopic astigmatism. J Cataract Refract Surg.

[30] Reinstein DZ, Gobbe M, Archer TJ (2013). Coaxially sighted corneal light reflex versus entrance pupil center centration of moderate to high hyperopic corneal ablations in eyes with small and large angle kappa. J Refract Surg.

[31] de Ortueta D, Arba-Mosquera S (2017). Laser in situ keratomileusis for high hyperopia with corneal vertex centration and asymmetric offset. Eur J Ophthalmol.

[32] Chan CC, Boxer Wachler BS (2006). Centration analysis of ablation over the coaxial corneal light reflex for hyperopic LASIK. J Refract Surg.

[33] Karhanová M, Pluháček F, Mlčák P, Vláčil O, Šín M, Marešová K (2015). The importance of angle kappa evaluation for implantation of diffractive multifocal intra-ocular lenses using pseudophakic eye model. Acta Ophthalmol.

[34] Fu Y, Kou J, Chen D (2019). Influence of angle kappa and angle alpha on visual quality after implantation of multifocal intraocular lenses. J Cataract Refract Surg.

[35] Liu Y, Gao Y, Liu R (2020). Influence of angle kappa-customized implantation of rotationally asymmetric multifocal intraocular lens on visual quality and patient satisfaction. Acta Ophthalmol.

[36] Barreto J Jr, Netto MV, Cigna A, Bechara S, Kara-José N (2006). Precision of higher order aberration repeatability with NIDEK OPD-scan retinoscopic aberrometry. J Refract Surg.

[37] Burakgazi AZ, Tinio B, Bababyan A, Niksarli KK, Asbell P (2006). Higher order aberrations in normal eyes measured with three different aberrometers. J Refract Surg.

[38] Koh S, Inoue R, Maeno S, Mihashi T, Maeda N, Jhanji V, Nishida K (2022). Characteristics of higher-order aberrations in different stages of keratoconus. Eye Contact Lens.

[39] Rocha KM, Nosé W, Bottós K, Bottós J, Morimoto L, Soriano E (2007). Higher-order aberrations of age-related cataract. J Cataract Refract Surg.

[40] Hashemi H, Khabazkhoob M, Jafarzadehpur E, Yekta A, Emamian MH, Shariati M, Fotouhi A (2015). Higher order aberrations in a normal adult population. J Curr Ophthalmol.

[41] Kandel S, Chaudhary M, Mishra SK (2022). Evaluation of corneal topography, pachymetry and higher order aberrations for detecting subclinical keratoconus. Ophthalmic Physiol Opt.

[42] Tonn B, Klaproth OK, Kohnen T (2014). Anterior surface-based keratometry compared with Scheimpflug tomography-based total corneal astigmatism. Invest Ophthalmol Vis Sci.

[43] Naeser K, Savini G, Bregnhøj JF (2018). Age-related changes in with-the-rule and oblique corneal astigmatism. Acta Ophthalmol.

